# First molecular characterization of poxviruses in cattle, sheep, and goats in Botswana

**DOI:** 10.1186/s12985-021-01634-9

**Published:** 2021-08-14

**Authors:** Boitumelo Magret Modise, Tirumala Bharani Kumar Settypalli, Tebogo Kgotlele, Dingrong Xue, Kebonyemodisa Ntesang, Kago Kumile, Ivancho Naletoski, John Frederick Nyange, Carter Thanda, Kenny Nametso Macheng, Chandapiwa Marobela-Raborokgwe, Gerrit Johannes Viljoen, Giovanni Cattoli, Charles Euloge Lamien

**Affiliations:** 1 Botswana National Veterinary Laboratory, Private Bag 0035, Gaborone, Botswana; 2 Animal Production and Health Section, Animal Production and Health Laboratory, Joint FAO/IAEA Division of Nuclear Techniques in Food and Agriculture, Department of Nuclear Sciences and Applications, International Atomic Energy Agency, Vienna International Centre, PO Box 100, 1400 Vienna, Austria; 3 Academy of National Food and Strategic Reserves Administration, Beijing, China

**Keywords:** Lumpy skin disease virus, Pseudocowpox virus, Orf virus, RPO30, GPCR, B2L gene, Botswana

## Abstract

**Background:**

Poxviruses within the *Capripoxvirus*, *Orthopoxvirus*, and *Parapoxvirus* genera can infect livestock, with the two former having zoonotic importance. In addition, they induce similar clinical symptoms in common host species, creating a challenge for diagnosis. Although endemic in the country, poxvirus infections of small ruminants and cattle have received little attention in Botswana, with no prior use of molecular tools to diagnose and characterize the pathogens.

**Methods:**

A high-resolution melting (HRM) assay was used to detect and differentiate poxviruses in skin biopsy and skin scab samples from four cattle, one sheep, and one goat. Molecular characterization of capripoxviruses and parapoxviruses was undertaken by sequence analysis of RPO30 and GPCR genes.

**Results:**

The HRM assay revealed lumpy skin disease virus (LSDV) in three cattle samples, pseudocowpox virus (PCPV) in one cattle sample, and orf virus (ORFV) in one goat and one sheep sample. The phylogenetic analyses, based on the RPO30 and GPCR multiple sequence alignments showed that the LSDV sequences of Botswana were similar to common LSDV field isolates encountered in Africa, Asia, and Europe. The Botswana PCPV presented unique features and clustered between camel and cattle PCPV isolates. The Botswana ORFV sequence isolated from goat differed from the ORFV sequence isolated from sheep.

**Conclusions:**

This study is the first report on the genetic characterization of poxvirus diseases circulating in cattle, goats, and sheep in Botswana. It shows the importance of molecular methods to differentially diagnose poxvirus diseases of ruminants.

## Background

Poxviruses exist throughout the world and are responsible for several economically significant zoonotic diseases affecting humans, wildlife, farming animals, and domestic animals [1, 2]. Generally, poxviruses are epitheliotropic and may cause localized cutaneous lesions or generalized lesions of skin, organs and tissues (respiratory and digestive tract mucosa, lungs and kidney). Skin lesions can be clinically misdiagnosed as other cutaneous diseases. Economically, poxvirus diseases cause losses due to damage to the skin, decreased milk and meat production and trade restrictions in addition to morbidity and mortality [3, 4].

Poxviruses are complex, linear, enveloped, double-stranded DNA viruses with large genomes of 130–360 kb in length [5, 6]. They belong to the *Poxviridae* family, which is divided into two subfamilies: *Entomopoxvirinae*, which infect invertebtates, and *Chordopoxvirinae,* which infect vertebrates [7]. The *Chordopoxvirinae* subfamily comprises 18 genera: *Orthopoxvirus*, *Parapoxvirus*, *Avipoxvirus*, *Capripoxvirus*, *Leporipoxvirus*, *Suipoxvirus*, *Molluscipoxvirus*, *Cervidpoxvirus*, *Crocodyli*d*poxvirus*, *Yatapoxvirus*, *Centapoxvirus*, *Macropopoxvirus*, *Mustelpoxvirus*, *Oryzopoxvirus*, *Pteropopoxvirus*, *Salmonpoxvirus*, *Sciuripoxvirus* and *Vespertilionpoxvirus* [8].

Among the *Chordopoxvirus* subfamily, are genera that affect livestock ruminants, some of which are capable of infecting the same animal species creating a challenge for clinical diagnosis. They include *Capripoxvirus* which is comprised of goatpox virus (GTPV), sheeppox virus (SPPV) and LSDV and *Parapoxvirus*, which includes ORFV, PCPV and bovine papular stomatitis virus (BPSV). Poxviruses can be transmitted through small abrasions of the skin (e.g.,ORFV), directly or indirectly from contaminated aerosols in the environment through the respiratory tract (e.g., SPPV and GTPV), and potential mechanical transmission by biting arthropods (e.g., LSDV and SPPV) [9–12].

In Botswana, poxvirus diseases in ruminants have received little attention, with no further characterization methods performed. The first publication on poxvirus diseases in Botswana was on ORFV in goats [13], without molecular characterization of the virus. Lumpy skin disease was first observed in 1943 following an outbreak in Ngamiland in northern Botswana. Since then, the disease has spread to most districts in the country [14]. In Botswana, LSD is controlled through vaccination with live attenuated vaccines; LSDV Neethling strain (Onderstepoort Biological Products; OBP, South Africa) and attenuated South African LSDV field isolate (Lumpyvax, MSD Animal Health-Intervet, South Africa). Even though LSD has been endemic and widespread in Botswana for many years, there is no genetic information available on LSDVs circulating in Botswana.

This study describes the clinical presentation, molecular detection, and molecular characterization of poxvirus diseases from clinical samples. We report the first confirmed case of pseudocowpox in Botswana, and the first molecular characterization of LSDV, ORVF, and PCPV in the country.

## Methods

### Study areas and sample collection

**Fig. 1:**
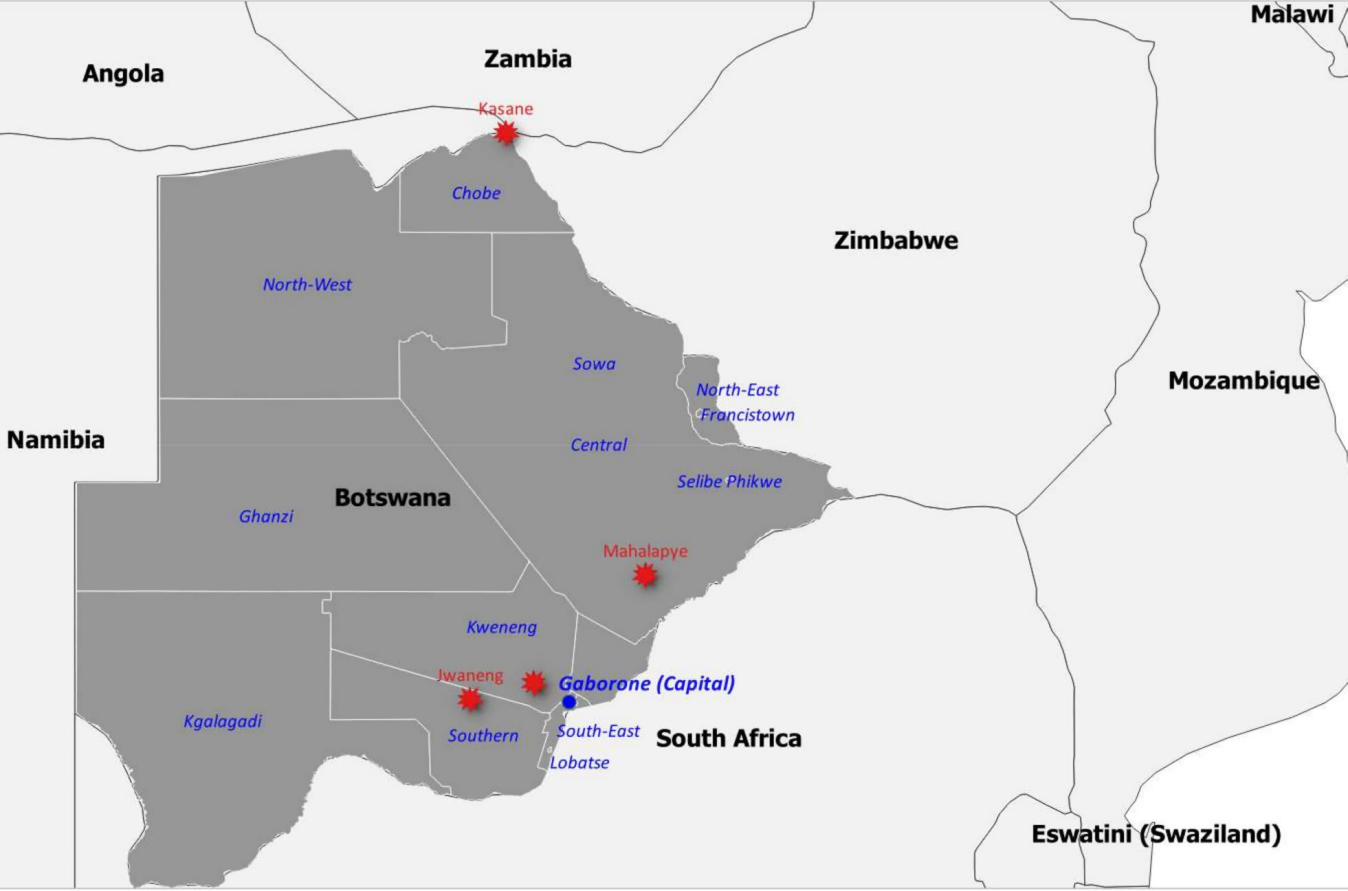
Map of Botswana showing the location of where samples were collected (red stars)

Six samples (three skin biopsies, one skin crust, one lip skin scab as well as one wart) from cattle, sheep and goat from Chobe, Central, Kweneng and Southern districts (Table 1 and Fig. 1) were used in this study. Chobe district in Northern Botswana has a high density of wildlife that regularly interact with livestock. The Central, Southern and Kweneng districts have a low density of wildlife and minimal to no contact with livestock.

**Table 1:** Information on the samples and animals from which specimens were collected for this study

Sample ID	District	Sub-district	Crush (Countrside/urban)	Year samples received	Host	Age	Gender	Breed	Type of samples
BOT_BOV/2010/6389	Central	Mahalapye	Tobela (Peri urban)	2010	Cattle	Adult	F	Sharolin	Skin crust
BOT_BOV/2016/172	Chobe	Kasane/Kavimba	Maruza (Countryside)	2016	Cattle	Information not available	Information not available	Local Tswana	Skin biopsy
BOT_OV/2017/158	Southern	Jwaneng	Sekwele (Countryside)	2017	Sheep	Information not available	Information not available	Dorper	Lip skin scab
BOT_BOV/2017/1657	Kweneng	Molepolole	Prison Farm (Peri urban)	2017	Cattle	Adult	F	Brown swiss	Skin biopsy
BOT_BOV/2019/246	Central	Mahalapye	Information not available	2019	Cattle	Information not available	Information not available	Information not available	Skin biopsy
BOT_CAP/2019/7419/74	Chobe	Kasane	Kachikau (Countryside)	2019	Goat	1 year	F	Local Tswana	Udder with warts

Samples were submitted by field personnel to the Botswana National Veterinary Laboratory (BNVL) for routine testing. Initially, one sample from 2010, one from 2016 and two from 2017 were tested by histopathological examination and the findings were suggestive of poxvirus infections (Table 3). In 2019, the same four samples and two additional samples from 2019 suspected of poxvirus infections were tested using a recently developed High Resolution Melting (HRM) assay for the simultaneous detection and differentiation of eight poxviruses of medical and veterinary importance, to confirm the poxvirus infections.

Epidemiological information including clinical signs and attempted clinical diagnosis were collected from original sample submission forms that accompanied the samples submitted to BNVL.

### Sample preparation and DNA extraction

The samples were cut with a scalpel blade into small pieces and homogenized (10% w/v) in sterile phosphate buffered saline (PBS), then centrifuged at 2500 rpm for 10 min to collect the supernatant. DNA was extracted from 200 µl of the supernatant using DNeasy Blood and Tissue kit (Qiagen) following the manufacturer’s instructions. The DNA was eluted in 80 µl elution buffer, then kept at − 20 °C until further use.

### Molecular detection and genotyping

The extracted DNA was tested using an HRM multiplex real-time assay for the simultaneous detection and differentiation of eight poxviruses of medical and veterinary importance [15]. The assay is based on high-resolution melting curve analysis of PCR amplicons produced using genus specific primer pairs and double stranded DNA binding dye, and exploits the differences in fragment size and GC content for discrimination. The method generates three well separated melting regions for each genus (*Orthopoxvirus, Capripoxvirus*, and *Parapoxvirus)* and provides additional genotyping of the viruses within each of the three genera (cowpox virus and camelpox virus in the *Orthopoxvirus* genus; GTPV, SPPV, and LSDV in the *Capripoxvirus* genus; ORFV, PCPV, and BPSV in the *Parapoxvirus* genus).

The PCR was set up in a 20 μl reaction volume containing 1 × SsoFast™ EvaGreen® Supermix (Bio-Rad), equal concentration (200 nM) of each of the forward and reverse primers (Table 2), and 2 μl of sample DNA. Each run included positive control plasmids representing each of the eight pathogens, and a negative control comprising nuclease-free water. The positive control plasmids used were Capripoxviruses (GTPV-Denizli, SPPV-Denizli, and LSDV-Ismalia), Orthopoxviruses (CMLV- Hadow/01/2012 and CPXV-72/93), and parapoxviruses (ORFV- DZ C-1, PCPV- 2200/12 and BPSV- Stamm M1) provided by IAEA. The PCR reactions and melting curve analysis were performed on the CFX96 Touch Real-Time PCR Detection System (Bio-Rad Laboratories), as previously described with slight modifications [15]. Briefly, an initial denaturation step at 95 °C for 4 min, followed by 40 cycles at 95 °C for 1 s, 59 °C for 5 s and 70 °C for 5 s. The PCR products were then denatured at 95 °C for 30 s, cooled down at 65 °C for 60 s, and melted from 65 °C to 85 °C with an increment of 0.2 °C every ten seconds and a continuous data acquisition. Data was analyzed using the CFX Maestro Software (Bio-Rad) and the Precision Melt Analysis Software (Bio-Rad).

**Table 2:** Primers used in this study for the HRM assay and sequencing

Method	Primer name	Primer sequence	Amplicon size	Target and references
HRM	CaPV-HRM-For	TCCTGGCATTTTAAGTAATGGT	100	Capripoxviruses [15]
	CaPV-HRM-Rev	GTCAGATATAAACCCGGCAAGTG		
	PPV-HRM-For	TCGAAGATCTTGTCCAGGAAG	112	Parapoxviruses [15]
	PPV-HRM-Rev	CCGAGAAGATCAACGAGGTC		
	OPV-HRM-For	AGGACTAGCCGCGGTAACTTT	56	Orthopoxviruses [15]
	OPV-HRM-Rev	ACAAGATAGAAGCGATGGATACTT		
Sequencing	CpGPCR-OL1F	TGAAAAATTAATCCATTCTTCTAAACA	617	Capripoxviruses [18]
	CpGPCR-OL1R	TCATGTATTTTATAACGATAATGCAAA		
	CpGPCR-OL2F	TTAGCGGTATAATCATTCCAAATA	603	
	CpGPCR-OL2R	GCGATGATTATGATGATTATGAAGTG		
	CpGPCR-OL3F	CACAATTATATTTCCAAATAATCCAA	684	
	CpGPCR-OL3R	TGTACATGTGTAATTTTAATGTTCGTA		
	CpRPO30-OL1F	CAGCTGTTTGTTTACATTTGATTTTT	554	
	CpRPO30-OL1R	TCGTATAGAAACAAGCCTTTAATAGA		
	CpRPO30-OL2F	TTTGAACACATTTTATTCCAAAAAG	520	
	CpRPO30-OL2R	AACCTACATGCATAAACAGAAGC		
	ORFV-B2Lf-For	GACCTTCCGCGCTTTAATTT	1210	Parapoxviruses [19]
	ORFV-B2Lf-Rev	CCCGCCTGCTAAAAGACT		

### Sequencing

The G-protein-coupled chemokine receptor (GPCR) [16], and the 30 kDa DNA-dependent RNA polymerase subunit (RPO30) [17] genes of the *Capripoxviruses* were amplified using primers [18] in Table 2. The PCR reaction was performed in a reaction volume of 20 µl containing 500 nM of each of the forward and reverse primers, 0.2 mM of dNTPs, 1 × buffer (Qiagen), 2.5 U of Taq DNA polymerase (Qiagen), and 2 µl template DNA. The amplification consisted of an initial denaturation at 95 ºC for 4 min followed by 35 cycles of 95 ºC for 40 s, 55 ºC for RPO and 56 ºC for GPCR for 30 s, and 72 ºC for 1 min, and a final extension step at 72 ºC for 7 min.

For the *Parapoxviruses,* the partial B2L gene was amplified by PCR in a reaction volume of 25 μl containing 500 nM of each of the primers [19] in Table 2, two mM dNTPs, 1X PCR Buffer (Qiagen), 2.5 U Taq polymerase (Qiagen) and five μl template DNA. The cycling conditions were: initial denaturation at 95 °C for 5 min, followed by 35 cycles at 95 °C for 50 s, 52 °C for 60 s and 72 °C for 90 s, and a final extension at 72 °C for 7 min.

All PCR products were separated by electrophoresis on a 1.5% agarose gel at 100 V for 1 h. The positive PCR products were purified using the Wizard SV Gel and PCR clean-up system kit (Promega) according to the manufacturer’s instructions, then sequenced commercially by LGC Genomics (Germany). The sequences were edited and assembled using Vector NTI Advance™11.5 software (Invitrogen, Carlsbad, CA, USA). All sequences were submitted to GenBank.

### Phylogenetic analysis

For phylogenetic reconstructions of the RPO30, GPCR, and B2L gene trees, multiple sequence alignments of the nucleotides sequences were performed separately for each gene using the muscle algorithm and the codon option implemented in MEGA7 [20]. Additional sequences of the RPO30 and GPCR gene for CaPVs (LSDVs, GTPVs, and SPPVs) and the B2L gene for parapoxviruses (ORFV, PCPV, and BPSV), were retrieved from GenBank and included for comparative analyses.

The sequence alignment FASTA file was converted into a Nexus format file using Seaview programme version 4.7 [21]. The Bayesian phylogenetic inference was performed with BEAST. First, the BEAUti module was used to generate BEAST files using the TN93 + G nucleotide substitution and a UPGMA starting tree. The Markov Chain Monte Carlo method was run with BEAST for 10,000,000 generations with a sample taken each 10,000 generations. The TRACER program was used to inspect the log files and determine the optimum number of burn-in based on the Effective Sample Sizes (ESS > 200). TreeAnnotator was used to generate the Maximum Clade Credibility (MCC) after discarding the 2% burn-in. The tree was visualized with the associated meta-data using the ggtree package in R version 3.5.2 [22]. Additionally, for the GPCR tree, the multiple sequence alignment file of the nucleotide sequences was imported and a slice of the alignment, between positions 80 and 120, was visualized together with the tree [22].

## Results

### Clinical diagnosis and molecular detection

It appeared that most animals had lesions suggestive either of poxvirus infections, and sometimes, papillomavirus infections (Table 3).

**Table 3:** Summary of clinical signs, attempted clinical diagnosis and molecular findings of the different poxviruses

Sample ID	Case history (clinical signs)	Attempted clinical diagnosis	Histopathology findings	Molecular findings	Sequenced gene (s) (Accession number)
BOT_BOV/2010/6389	Recumbent, bleeding wounds on the udder, mouth, anus, and legs	Papillomatosis	Sample not suitable for testing	PCPV positive	B2L (MW748473)
BOT_BOV/2016/172	Cattle developing skin nodules all over the body	LSD	Proliferative dermatitis Suggestive of LSD	LSDV positive	RPO30 (MW748474); GPCR (MW748477)
BOT_OV/2017/158	Lesions on and around mouth and buccal cavity	Orf	Referred to laboratory outside Botswana but results were not received	ORFV positive	B2L (MW748471)
BOT_BOV/2017/1657	Cow with skin bumps and lameness	LSD	Proliferative dermatitis with collegenolysis suggestive of LSD	LSDV positive	RPO30 (MW748475); GPCR (MW748478)
BOT_BOV/2019/246	Lesions on the skin, nasal discharge, and lacrimation	LSD	Test not done	LSDV positive	RPO30 (MW748476); GPCR (MW748479)
BOT_CAP/2019/74	Udder of a dead goat with papilloma warts like/ orf infection on udder	Orf, Papilloma warts	Test not done	ORFV positive	B2L (MW748472)

**Fig. 2:**
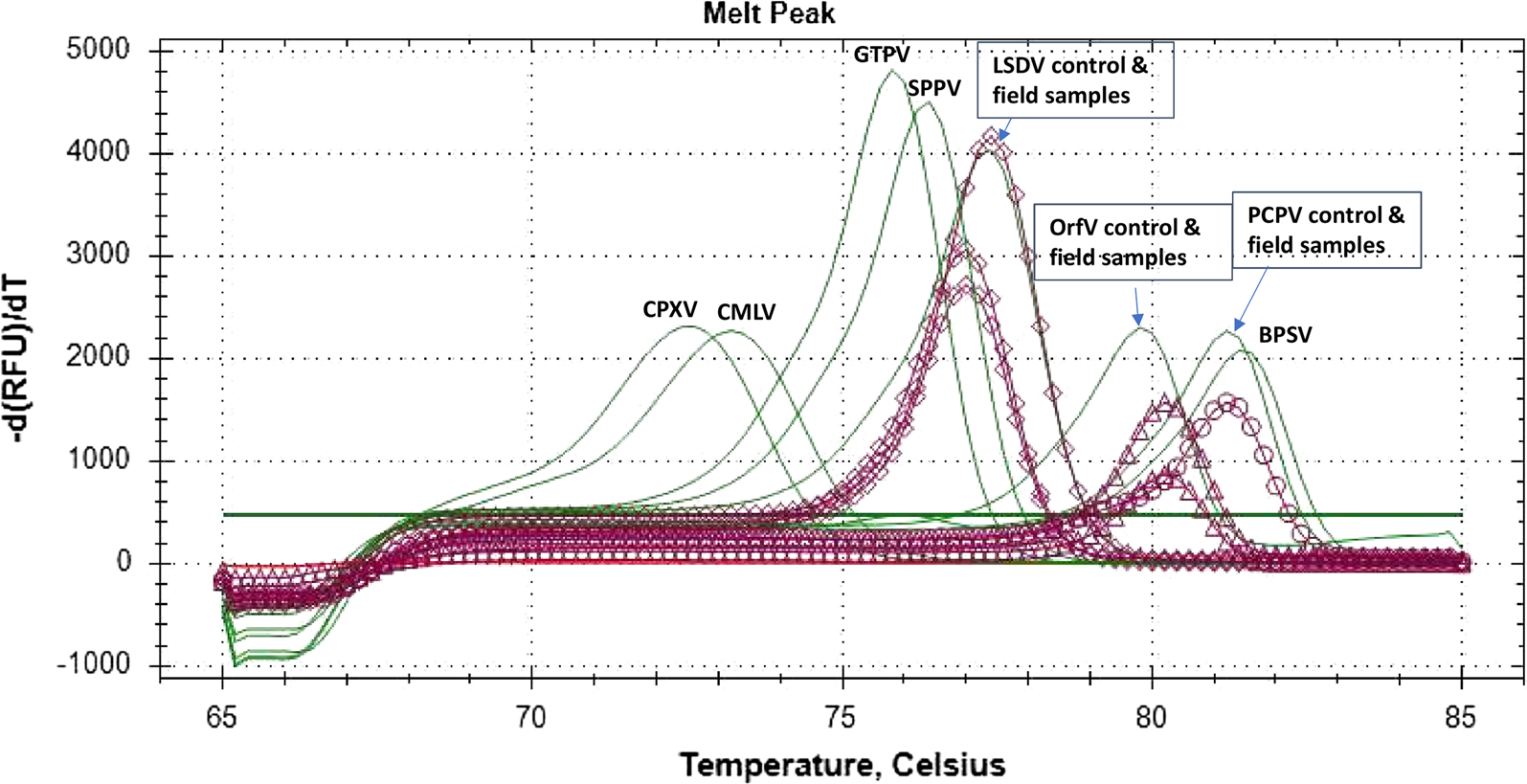
HRM detection of poxvirus diseases in cattle, goats, and sheep samples from Botswana. The positive control for each of the eight poxviruses displayed a unique melting peak, shown in green color. One cattle and three cattle samples clustering with PCPV and LSDV, respectively, and one goat and one sheep samples clustering with ORFV are shown in purple colour

As summarised in Table 3, PCPV DNA was detected in one out of four cattle samples (BOT_BOV/2010/6389) and LSDV DNA in three out of four cattle samples (BOT_BOV/2016/172, BOT_BOV/2017/1657, and BOT_BOV/2019/246) using the HRM real-time PCR assay. ORFV DNA was detected in both sheep and goat samples, respectively (BOT_OV/2017/158 and BOT_CAP/2019/74), with Orthopoxvirus DNA absent in all samples. Figure 2 shows the melting peaks corresponding to PCPV, LSDV, and ORFV in the Botswana samples.

### Molecular characterization and phylogenetic analysis

Two fragments for the RPO30 gene (554 bp and 520 bp) and three fragments for the GPCR gene (617 bp, 603 bp, and 684 bp) were successfully amplified and sequenced in all three LSDV positive samples (BOT_BOV/2016/172, BOT_BOV/2017/1657, and BOT_BOV/2019/246). Similarly, an approximately 1210 bp fragment of the B2L gene was amplified and sequenced in the remaining three samples that were positive for PCPV (BOT_BOV/2010/6389) and ORFV (BOT_OV/2017/158, and BOT_CAP/2019/74). The complete RPO30 and GPCR genes and partial B2L gene sequences were submitted to GenBank database under accession numbers MW748471 to MW748479.

**Fig. 3:**
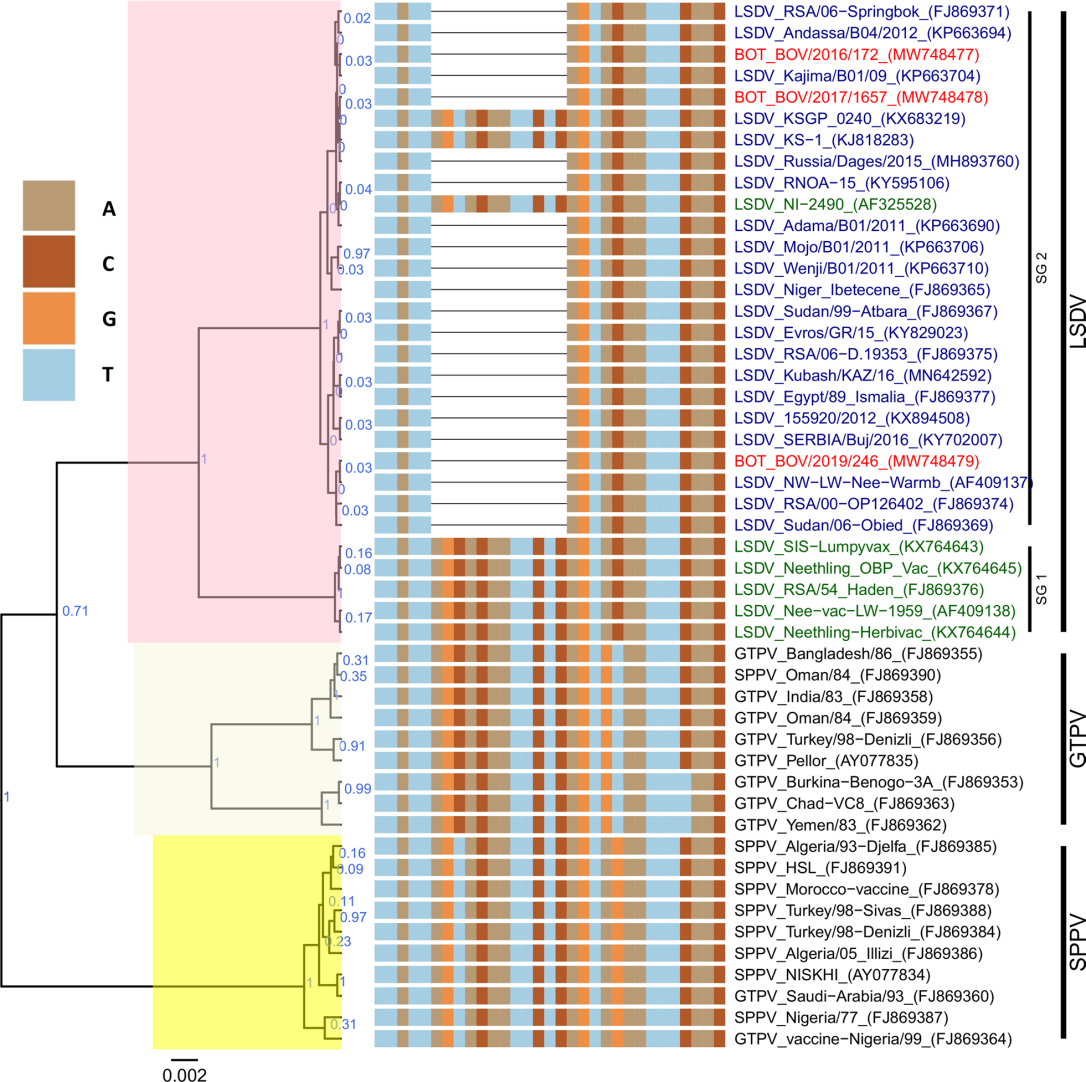
Maximum clade credibility (MCC) tree based on the complete GPCR gene sequences of Capripoxviruses. Only the portion of the alignment between positions 80 and 110 is shown. The posterior probabilities are plotted as respective nodes labels. LSDVs from Botswana are highlighted in red. Representive reference sequences, LSDV (twenty-seven), GTPV (nine) and SPPV (ten) were retrieved from GenBank and included in the phylogenetic tree. LSDV collected before 1960 are shown is dark green, those after 1960 are shown in dark blue, and other capripoxviruses (SPPV and GTPV) are in black

**Fig. 4:**
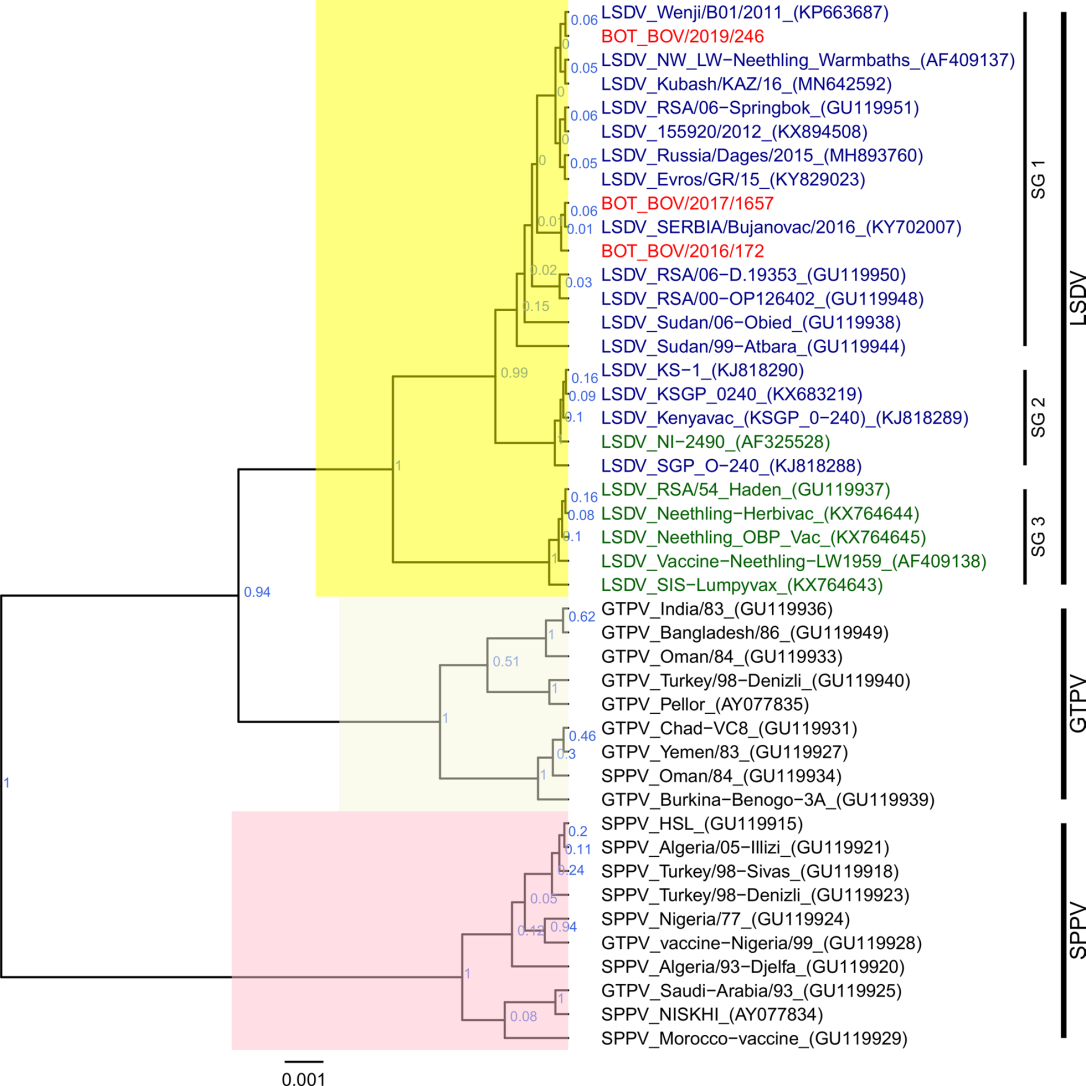
Maximum clade credibility tree based on the complete RPO30 complete gene sequences of capripoxviruses. The posterior probabilities are plotted as respective nodes labels. LSDVs from Botswana are highlighted in red. Representive reference sequences, LSDV (twenty-two), GTPV (nine) and SPPV (ten) were retrieved from GenBank and included in the phylogenetic tree. LSDV collected before 1960 are shown is dark green, those after 1960 are shown in dark blue, and other capripoxviruses (SPPV and GTPV) are in black

In both GPCR and RPO30 gene phylogenetic trees, all three cattle samples from Botswana (BOT_BOV/2016/172, BOT_BOV/2017/1657, and BOT_BOV/2019/246) clustered within the LSDV group (Figs. 3 and 4). For the GPCR gene, the LSDV group was further subdivided into two subgroups: the first subgroup included the LSDVs from Botswana, and field LSDVs from Europe, the Middle East and Africa, and LSDV KS1 derived vaccinal viruses. The second subgroup consisted of LSDV Neethling-like viruses, and the historical LSDV Haden 1959 from South Africa (Fig. 3).

The multiple sequence alignments of the GPCR gene showed that all the Botswana LSDVs had sequences identical to each other, and presented a 12-nucleotide deletion found in common field LSDV isolates (Fig. 3).

For the RPO30 gene (Fig. 4), LSDV isolates produced 3 sub-groups: the first one consisted of the Botswana LSDVs and common field LSDVs encountered in Africa, the Middle East and Europe. The second subgroup contained LSDV KS1-like viruses and LSDV NI-2490, while the third consisted of LSDV Neethling like viruses and the historical LSDV Haden 1959 (Fig. 4).

The RPO30 gene sequences were identical for all the Botswana LSDVs and identical to common field isolates, but different from the the vaccinal strains derived from KS1 and Neethling viruses. The Botswana isolates differed from LSDVs from Sudan, (GU119938) and (GU119944), by a single non synomymous nucleotide difference leading to amino acid substitutions at one position (T^14^ → N and D^102^ → G) respectively.

**Fig. 5:**
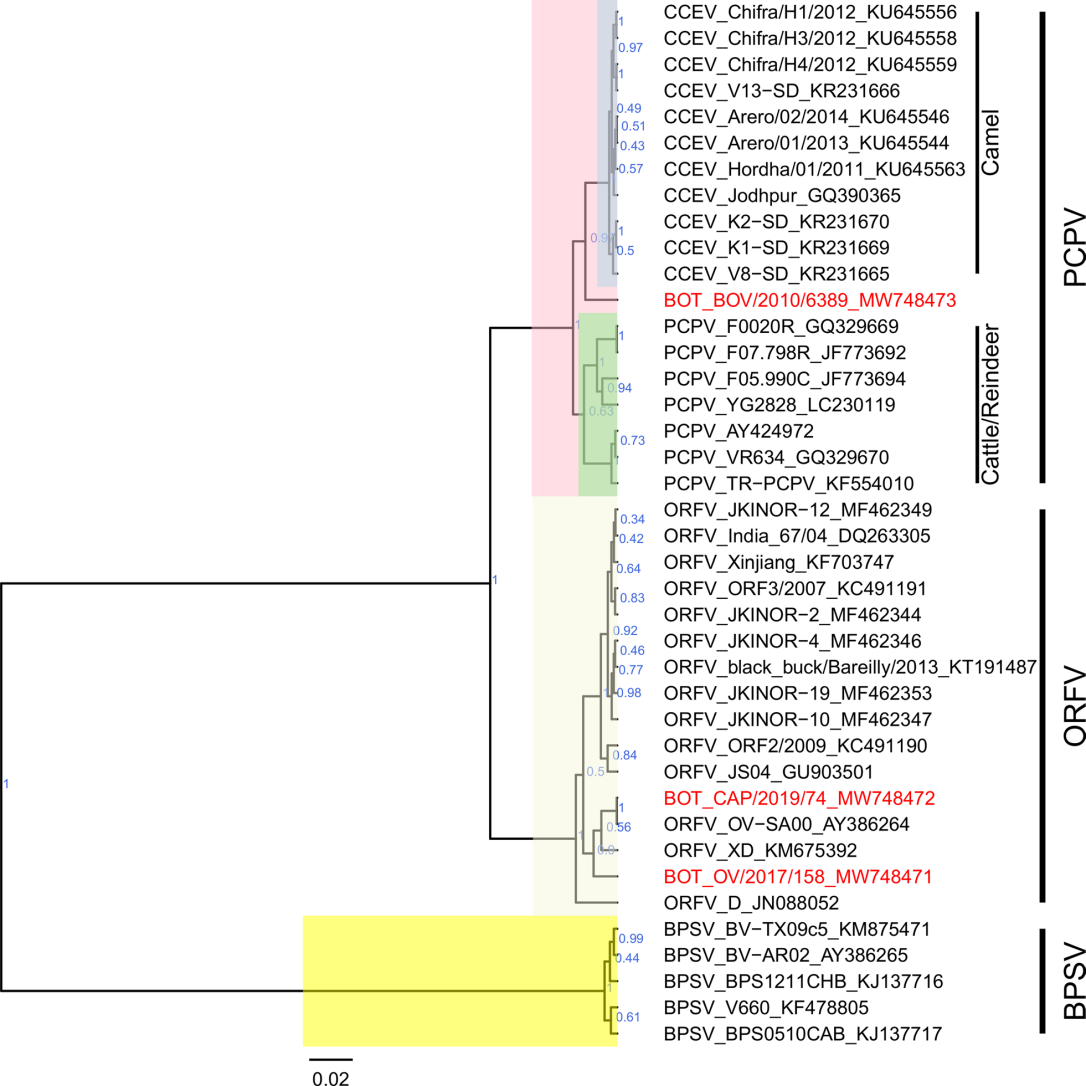
Maximum clade credibility tree based on the partial B2L gene sequences of parapoxviruses. The posterior probabilities are plotted as respective nodes labels. The cattle PCPV sequence and sheep and goat ORFV sequences from Botswana, are highlighted in blue. Representive reference sequences (black color), PCPV (eighteen) ORFV (fourteen) and BPSV (five) were retrieved from GenBank and included in the phylogenetic tree

On the phylogenetic tree of the B2L gene, the sequence of sample BOT_BOV/2010/6389 clustered with PCPVs (Fig. 5). Within the PCPV group, BOT_BOV/2010/6389 clutered between the B2L sequences of camel isolates and those of cattle/reindeer isolates. The B2L sequences of the two samples BOT_OV/2017/158 and BOT_CAP/2019/74, collected from small ruminants, clustered within the ORFV group (Fig. 5).

The BLAST results showed that the nucleotide identities of the Botswana PCPV, BOT_BOV/2010/6389, against published parapoxviruses ranged from 98.63%-98.97% with the highest identity (98.97%) to PCPV strains strain 3/07 (KF478804) isolated from cattle in Germany, and strain VR634 (GQ329670) collected from man infected by cattle in New Zealand.

The B2L sequence of ORFV BOT_CAP/2019/74 was 100% identical to that of strain OV-SA00 (AY386264) isolated from a goat in the USA. The multiple sequence alignments of the B2L gene revealed 24 nucleotide changes between the B2L gene of BOT_CAP/2019/74 and BOT_OV/2017/158.

## Discussion

This study presents the first confirmed case of PCPV in Botswana and the first molecular characterization of poxviruses affecting sheep, goats and cattle in the country from animals presenting clinical lesions consistent with poxvirus infections. The DNA of LSDV (3 samples), PCPV (1 sample), and ORFV (2 samples) were detected in clinical samples from 2010, 2016, 2017, and two recent samples from 2019 using an HRM assay for the differential diagnosis of poxvirus infections [15]. It is worth noting that the status of the 2010 sample was previously undetermined, until the availability of this HRM assay which showed the presence of PCPV. Hence, the HRM assay has enabled the correct identification of LSDV and PCPV in cattle and ORFV in small ruminants in a single convenient assay [15].

The attempted clinical diagnosis of the poxvirus diseases were mostly consistent with the results of the HRM assay for LSDV and ORFV. The LSD suspected cattle had typical clinical signs that included skin nodules/bumps/lesions [23, 24] and orf suspected small ruminants had papilloma warts on the udder and/ or lesions on and around the mouth and buccal cavity as previously described [25, 26]. However, in this study, the lesions decribed for the PCPV positive animal were mostly suggestive for papillomavirus infections, though, the lesions on the udder of cattle are also common in pseudocowpox infections [27, 28].

For all samples, the sequencing data was in full agreement with the HRM data. The RPO30 and GPCR gene sequences of the Botswana LSDV samples were identical even through they were collected from three different years, 2016, 2017 and 2019, and from 3 different geographic locations (Central, Chobe and Kweneng districts). This suggests that the virus is well conserved and that the same strain is responsible for all cases.

Moreover, the Botswana LSDV sequences presented similar features to common field isolates of LSDV encountered in Africa, Europe and Asia, including the presence of the 12-nucleotide deletion in their GPCR gene [16, 18, 29–31].

The existence of PCPV in cattle has been reported worldwide [32–35]. In Africa, the disease has recently been reported in Zambia [36] and now in Botswana (this study). Interestingly, the phylogenetic analysis revealed that the newly sequenced PCPV from Botswana was distinct from common cattle PCPVs encountered elsewhere, behaving like an intermediate isolate between cattle isolates of PCPV and camel isolates of PCPV (known as CCEV). This finding is uncommon, as most isolates recovered from cattle usually clustered with the cattle/reindeer isolates. For instance, PCPVs that were recently reported in Zambia presented substantial sequence variation within the same herd and during the same outbreak events in 2017 and 2018, but were all clustering with the cattle isolates of PCPVs [36]. The unique genetic feature of the Botswana PCPV suggests that by the time the case was recorded in 2010, PCPV was already well established in the country.

The path of introduction of the PCPV strain to Botswana is unknown and requires further investigation. Additional genetic analysis of the PCPV strain is also required to determine if it is genetically distinct enough to be classified as cattle,reindeer or camel variant of PCPV virus.

The phylogenetic tree based on the B2L gene sequence, and the scrutiny of the multiple sequence alignment showed that the sample collected in sheep in 2017 in northern Botswana differed from that of the sample collected in goat in 2019 in the southern part of the country, suggesting that at least two different strains of ORFV are circulating in the country.

The caprine ORFV B2L sequence was very similar to an ORFV OV−SA00 (AY386264) isolate collected from a goat in the USA in 2000. The ovine ORFV sequence differed from most ovine sequences, but displayed 99.6% amino acid similarity to isolate FJ-ZX (KC568400) collected from a goat in China in 2012.

## Conclusion

This paper reports the first molecular detection and characterization of poxvirus diseases circulating in sheep, goat and cattle in Botswana. It shows the importance of molecular methods for the differential diagnosis of poxvirus diseases in ruminants (the presence of LSDV and PCPV in cattle and ORFV in small ruminants). Based on our findings, we recommend the HRM assay be used as a rapid screening and confirmatory tool for poxvirus infections during disease investigations and epidemiological studies. PCPV is a zoonotic disease that farmers in Botswana have limited information on. Publication of this information coupled with improved public health awareness campaigns to local farmers will provide the necessary information about pseudocowpox virus to educate future recognition of disease and timely reporting and treatment. Considering PCPV’s risk of dissemination, strict hygiene measures should be applied to prevent transmission to humans when detected. Country-wide surveillance is recommended to determine the prevalence of LSDV and PCPV infections in cattle, ORFV in sheep and goats in Botswana, and to identify infection risks for other animals and humans. The correct identification of the etiologic agent of pox-like lesions in ruminants is essential to allow for proper pox disease management and control, and to improve veterinary interventions, including vaccination of non-infected surrounding herds in the case of LSD.

## Data Availability

DNA sequences generated and analyzed under the current study are available in GenBank under accession numbers MW748471, MW748472, and MW748473 (B2L gene), MW748474, MW748475, and MW748476 (RPO30 gene), MW748477, MW748478, and MW748479 (GPCR gene).

## Abbreviations

BNVL: Botswana National Veterinary Laboratory
BPSV: Bovine papular stomatitis virus
DNA: Deoxyribonucleic acid
GTPV: Goatpox virus
HRM: High resolution melting
LSDV: Lumpy skin disease virus
MCC: Maximum Clade Credibility
ORFV: Orf virus
PBS: Phosphate buffered saline
PCR: Polymerase chain reaction
PCPV: Pseudocowpox virus
SPPV: Sheeppox virus
